# Genome-Wide Association Study of Cuticle and Lipid Droplet Properties of Cucumber (*Cucumis sativus* L.) Fruit

**DOI:** 10.3390/ijms25179306

**Published:** 2024-08-28

**Authors:** Stephanie Rett-Cadman, Yiqun Weng, Zhangjun Fei, Addie Thompson, Rebecca Grumet

**Affiliations:** 1 Department of Horticulture, Graduate Program in Plant Breeding, Genetics and Biotechnology, Michigan State University, East Lansing, MI 48824, USA; rettstep@msu.edu; 2 Department of Plant and Agroecosystem Sciences, University of Wisconsin, Madison, WI 53706, USA; yiqun.weng@wisc.edu; 3 USDA-ARS Vegetable Crops Research Unit, Madison, WI 53706, USA; 4 Boyce Thompson Institute, Cornell University, Ithaca, NY 14853, USA; zf25@cornell.edu; 5 USDA-ARS Robert W. Holley Center for Agriculture and Health, Ithaca, NY 14853, USA; 6 Department of Plant, Soil and Microbial Sciences, Graduate Program in Plant Breeding, Genetics and Biotechnology, Michigan State University, East Lansing, MI 48824, USA; thom1718@msu.edu

**Keywords:** fruit surface, GWAS, SHINE1/WIN1, cucumber core collection, AAI domain-containing protein, cuticle, lipid droplets, oil bodies, cucurbits

## Abstract

The fruit surface is a critical first line of defense against environmental stress. Overlaying the fruit epidermis is the cuticle, comprising a matrix of cutin monomers and waxes that provides protection and mechanical support throughout development. The epidermal layer of the cucumber (*Cucumis sativus* L.) fruit also contains prominent lipid droplets, which have recently been recognized as dynamic organelles involved in lipid storage and metabolism, stress response, and the accumulation of specialized metabolites. Our objective was to genetically characterize natural variations for traits associated with the cuticle and lipid droplets in cucumber fruit. Phenotypic characterization and genome-wide association studies (GWAS) were performed using a resequenced cucumber core collection accounting for >96% of the allelic diversity present in the U.S. National Plant Germplasm System collection. The collection was grown in the field, and fruit were harvested at 16–20 days post-anthesis, an age when the cuticle thickness and the number and size of lipid droplets have stabilized. Fresh fruit tissue sections were prepared to measure cuticle thickness and lipid droplet size and number. The collection showed extensive variation for the measured traits. GWAS identified several QTLs corresponding with genes previously implicated in cuticle or lipid biosynthesis, including the transcription factor *SHINE1/WIN1*, as well as suggesting new candidate genes, including a potential lipid-transfer domain containing protein found in association with isolated lipid droplets.

## 1. Introduction

The surface of the cucumber fruit is covered by the cuticle, a hydrophobic layer that serves as a barrier to abiotic and biotic stresses. The functions of the cuticle can include the limitation of non-stomatal water loss, defense against pathogens and insects, and protection against drought and damage from UV radiation [1,2,3]. The functionality of the cuticle can influence shipping and handling procedures along with the longevity of the fruit in the market [4,5]. The cuticle comprises a complex matrix of cutin monomers and waxes; the substrates of these components are synthesized by epidermal cells and transported to the cell surface [1,3]. Waxes are derived from very-long-chain fatty acids, which can include structures such as alkanes, aldehydes, primary and secondary alcohols, ketones, and esters. Lipid droplets facilitate this process by storing some of the necessary lipid components, while their associated proteins conduct enzymatic reactions whose products are transported to the cell wall by microtubules to form the cutin matrix [6].

The cuticle provides mechanical support for fruit throughout development and can vary in composition and thickness between developmental ages [3,4]. In cucumber, deposition of the cuticle coincides with the fruit growth curve, where peak expression of cuticle-associated genes occurs during the exponential phase of fruit growth, around 8–12 days post-anthesis (dpa), and is largely complete by the end of exponential growth, at approximately 16 dpa [7,8]. Variation has also been observed in different varieties of cucumber; for example, North American pickling types tend to have thicker cuticles than Asian fresh market varieties [8,9].

External fruit quality traits can also be impacted by cuticle properties and can therefore influence consumer preferences, with glossy cucumber fruit often preferred. Variations in cucumber fruit glossiness are influenced by several genes. For instance, *CsCYP86B1*, identified within the QTL region, and *CsFSG1*, on chromosome 3, showed increased expression in high-gloss lines in early fruit development. The CsCYP86 subfamily of cytochrome P450s encodes fatty acid ω-hydroxylases that, in Arabidopsis, are involved in surface lipid polymer biosynthesis [10,11]. In cucumber, homologs of *ECERIFERUM 1* (*CsCER1*) and *ECERIFERUM 3* (*CsWAX2*) play important roles in alkane production and impact cutin and wax biosynthesis by reducing wax deposition and cutin composition, leading to increased fruit glossiness [12,13]. Similarly, *CsCER6* and *CsCER7* positively regulate wax accumulation in cucumber, which negatively impacts fruit glossiness because a higher wax load is associated with less glossy fruit [14]. In contrast, higher expression of *CsCER4* during early fruit development results in glossier fruit, likely due to differences in wax composition, where *CsCER4* increases primary alcohol production and decreases alkane formation [15]. In addition, *CsSEC23*, which encodes a component of the COPII vesicle transport complex, influences the transport of wax and cutin to the plasma membrane [16]. Mutations in *CsSEC23* lead to glossier fruit through alterations in the wax and cutin load and changes in the cuticle structure.

Transcriptional regulation of cuticle-associated traits can also impact fruit surface traits and account for some of the natural variations observed within cucumber fruit. The transcription factor *CsSHINE1/WAXINDUCER1* (*CsSHN1/WIN1*) contributes to variations in epidermal traits, such as cuticle thickness, cuticle intercalation between epidermal cells, epidermal cell shape, and size of lipid droplets within the epidermal cell layer [8]. *CsSHN1* is preferentially expressed in cucumber fruit peel, and its peak expression occurs during the cell expansion phase of fruit growth, coinciding with the period of peak cuticle deposition [7,8]. For cucumber plants grown on pumpkin rootstock, *CsWIN1* is methylated and upregulated, which, in turn, increases expression of wax biosynthetic genes including *CsCER1* and *CsCER4* [17]. The high expression of *CsWIN1* and other wax biosynthetic genes influences the content of wax load, resulting in an increase in wax esters and a decrease in alkanes. The higher wax ester content is associated with a glossier appearance of cucumber fruit [17]. The copy number variation of *CsSHN1/WIN1* is also associated with the degree of netting observed in cucumber fruit. Cucumber lines with multiple functional copies have been observed to have heavy skin netting, while those with only one functional copy experience only light skin netting, and loss-of-function mutants of *CsSHN1/WIN1* exhibit smooth (non-netting) skin [18]. Increased expression of the C_2_H_2_-type zinc finger transcription factor (CsZFP6/CsDULL) encoded by *CsDULL/CsGlossy Fruit* (*GLF1*) leads to an accumulation of wax and cutin in the peel. Loss of function of CsZFP6/CsDULL, decreases wax accumulation and leads to glossier fruit [19,20].

Also contained in the epidermal layer are lipid droplets, which consist of a neutral core surrounded by a phospholipid monolayer with embedded proteins. Lipid droplets are increasingly recognized as highly dynamic entities that potentially modulate intracellular metabolism and communication [21]. Lipid droplets can vary in their composition and assist in functions such as lipid metabolism, stress response, and the accumulation of specialized metabolites. Previous studies of cucumber fruit showed highly visible lipid droplets in the epidermal layer that varied in number and size depending on developmental age and variety [8]. While lipid droplets assist with cuticle formation, they continue to persist in the epidermis of cucumber fruit after cuticle deposition is largely complete and so may perform additional physiological roles [8].

There have been far fewer studies that examine the natural variation for cuticle and lipid droplet traits. Previous studies of seven species of tomato [22] and fifty accessions of pepper [23] examined the diversity in cuticle structure and composition but not the genetic factors contributing to this diversity. Using genome-wide association studies (GWAS) and genetic mapping in wax gourd [*Benincasa hispida* (Thunb) Cogn.], *BhWAX*, encoding a membrane-bound O-acyltransferase (MBOAT), has been proposed as the candidate gene responsible for mature fruit cuticular wax accumulation, while several QTLs contributing to cuticle thickness and density in Spanish peach landraces have been identified [24,25]. There is even less information regarding genetic factors regulating the size and number of lipid droplets. In Arabidopsis seeds, SEIPIN proteins contribute to the accumulation and size of lipid droplets, while oil-body-associated protein 1 (OBAP1) has been found to contribute to lipid droplet size [26,27]. 

While many genes contribute to cuticle biosynthesis, knowledge of these traits, as described above, primarily comes from studies involving biparental populations or candidate gene-based approaches that rely on prior knowledge from other species. In this work, we sought to examine natural variations in cuticle and lipid droplet features. The CucCAP project has made available genomic resources, including a cucumber core collection consisting of 388 accessions that account for >96% of the allelic diversity present in the cucumber collection of the United States National Germplasm System (NPGS) [28,29]. These lines were resequenced to a depth of 30–40× and ~2.5 million SNPs were identified [30]. This study utilized these recent genomic resources to identify new sources of variation and genetic components underlying cuticle and lipid droplet traits in cucumber.

## 2. Results

### 2.1. Diversity of Cuticle and Lipid Droplet Traits

**Figure 1:**
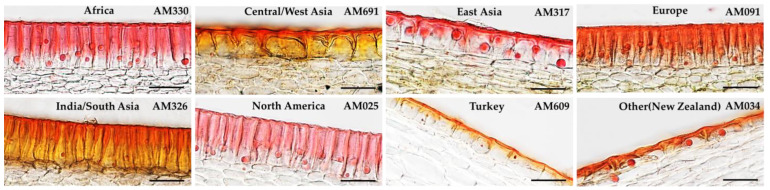
Examples of the diversity of cuticle and lipid droplet traits in the cucumber core collection. Fresh tissue sections of various accessions in the collection. Scale bar represents 50 µm.

**Figure 2:**
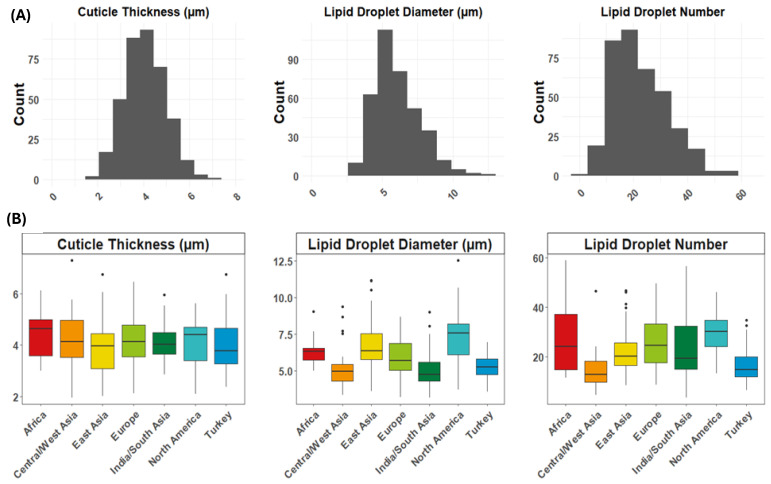
Distribution of cuticle and lipid droplet traits for fruit epidermis of the cucumber core collection (*n* = 374). (**A**) Trait distribution using best linear unbiased estimates (BLUEs) of accessions for combined data from 2019–2021. (**B**) Distribution of epidermal trait values based on region of origin. Geographic regions were assigned as per Wang et al. [29]. Values for each accession are based on measurements from three fruits per accession per year.

The CucCAP cucumber core collection (*n* = 374 lines for which we were able to collect phenotype data) showed great diversity for a variety of epidermal traits, including cuticle thickness and lipid droplet size and number (Figure 1 and Figure 2; Supplementary Table S1). All three traits showed a normal distribution, indicating quantitative inheritance (Figure 2A). Cuticle thicknesses ranged from 1.13 to 10.67 µm, with a 9-fold variation (Table 1). Lipid droplet diameters ranged from 2.06 to 12.82 µm, with a 6-fold variation. Lipid droplet number showed the greatest trait variability, with a range of 3 to 81 lipid droplets per accession, leading to a 27-fold variation. 

A set of 50 accessions was grown in all three seasons to assess trait reproducibility across seasons. Lipid droplet number and size were highly reproducible between years (*r* = 0.75 ***–0.82 *** and 0.68***–0.81 ***, respectively; Supplementary Table S2), but cuticle thickness was somewhat more variable between years (*r* = 0.31 *–0.46 ***). Consistent with the high reproducibility observed over seasons, lipid droplet diameter had a high broad sense heritability estimate of 0.70. Lipid droplet number and cuticle thickness had moderate broad-sense heritability estimates of 0.52 and 0.45, respectively (Table 1). Cuticle thickness was moderately correlated with droplet diameter (0.46 ***), but not with lipid droplet number (0.11). Lipid droplet number and diameter were moderately correlated (0.44 ***). The modest or low correlation among traits suggests differential regulation of these traits. Lipid droplet traits were highly variable among lines with different geographic origins (Figure 2B). Geographic regions were assigned as per Wang et al. [29] in accordance with their phylogenetic relationships. Accessions with smaller and fewer lipid droplets tended to originate from Central/West Asia, India/South Asia, and Turkey, while accessions from North America tended to have larger lipid droplets and a greater number of them. Although differences in cuticle thickness were not significant among the different geographic regions, the cucumber accessions with the lowest cuticle thickness most frequently originated from East Asia (43% of the accessions with cuticles < 3 µm were from East Asia; Supplementary Table S1). 

### 2.2. GWAS Analysis of Cuticle Thickness

**Figure 3:**
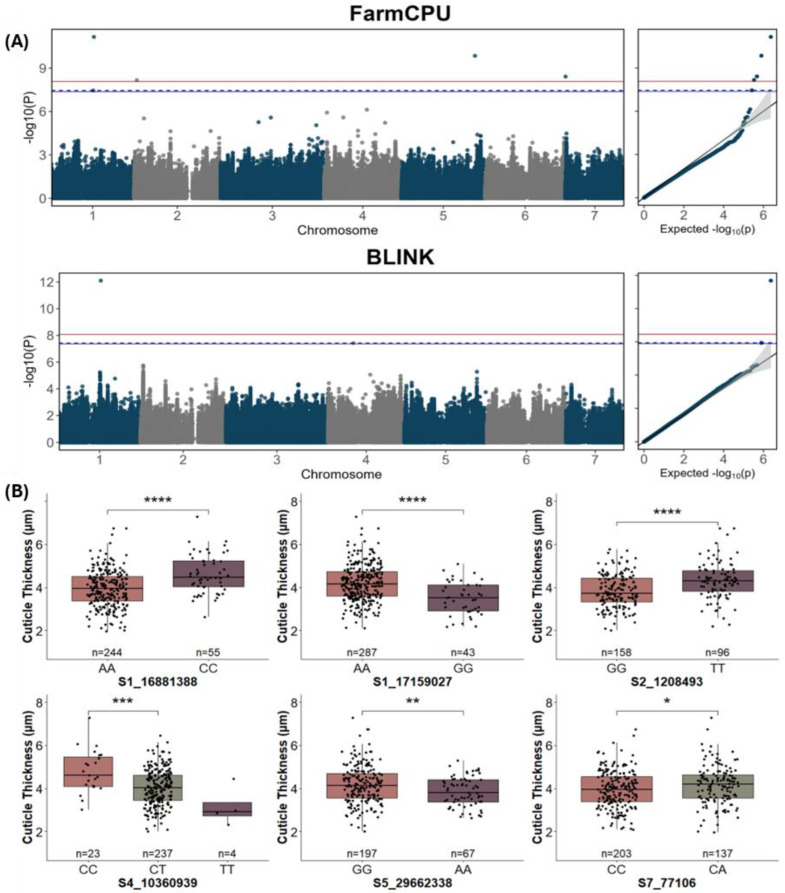
Manhattan, QQ, and allele effect plots for cuticle thickness for fruit from the cucumber core collection using BLUE values. (**A**) FarmCPU and BLINK models of GWAS. The blue and red lines represent Bonferroni-corrected *p*-values of 0.05 and 0.01, respectively; the dashed blue line represents FDR ≤ 0.05. (**B**) SNP markers with significant allelic effects. BLUE values were calculated from combined data from 2019–2021. *, **, ***, and **** represent *p* ≤ 0.05, 0.01, 0.001, and 0.0001, respectively. Heterozygotes were included if they were >10% of the population.

GWAS analysis was performed on the 367 accessions for which genotype and phenotype data were available. The results of GWAS analysis using FarmCPU and BLINK models [GAPIT 3.0” software [31] in R (version 4.2.2)] at a Bonferroni-corrected threshold of α = 0.05 and FarmCPU, BLINK, MLMM, MLM, and GLM models at a false discovery rate (FDR) of *p* ≤ 0.05 for cuticle thickness are illustrated in Figure 3A, Supplementary Figure S1, and Table 2. Six SNP markers significantly associated with cuticle thickness, located on chromosomes 1, 2, 4, 5, and 7, explained 2.05–23.43% of the phenotypic variation observed for this trait. One SNP, S1_17159027 (chromosome 1, position 17,159,027 bp; Gy14v2.1), was found to be significant by all the models (FarmCPU, BLINK, MLMM, MLM, GLM) tested using the Bonferroni-corrected threshold. At FDR ≤ 0.05, four of the six significant SNPs were identified by multiple models (Table 2). All identified SNPs showed significant allele effects for cuticle thickness (Figure 3B).

The majority of accessions in the core (>96%) were homozygous for either major or minor alleles at each nucleotide position. Only 45,415 of the set of 1.18 M SNPs (3.9%) had greater than 10% accessions that were heterozygous at any given position and, on average, each accession was homozygous at 93.8% of the SNP positions. A notable exception to this pattern was found in SNP S4_10360939, which was indicated to represent 23.43% of the variation by the BLINK model (Figure 3B, Table 2). The heterozygotes (“CT”) accounted for 89.8% of genotypes, where only four accessions were homozygous for the “T” allele. The phenotypic difference in cuticle thickness between the homozygotes was greater for this SNP than for the other SNPs for this trait. The heterozygous phenotype is an intermediate value between the homozygous allele phenotypes, indicating the additive effect of this allele. Another deviation occurred on chromosome 7, where SNP S7_77106 was identified by the FarmCPU model, as there were no accessions that were homozygous for the minor “A” allele, and heterozygous “CA” genotypes made up approximately 40% of the genotypes. The regions surrounding S4_10360939 and S7_77106 showed elevated rates of heterozygosity for distances of 8 kb and 4 kb, respectively. Whether the high level of heterozygosity may reflect a fitness advantage, either related to the cuticle trait, or due to linkage association with other traits, remains to be determined. 

**Figure 4:**
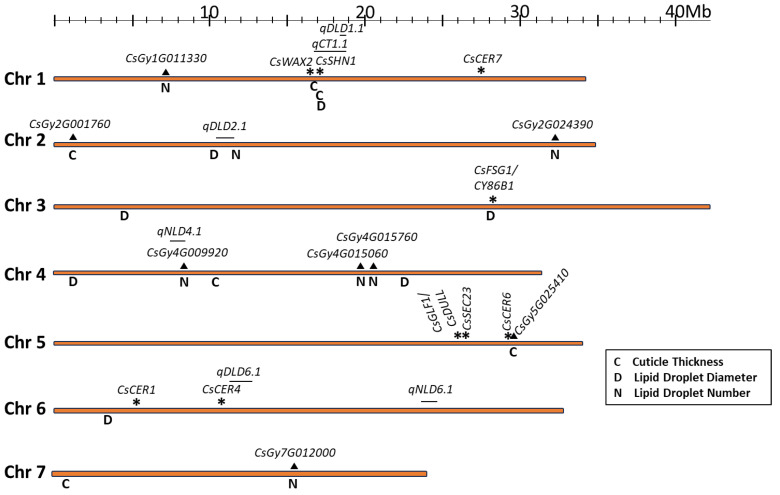
Chromosomal locations of significant SNPs identified by GWAS for cuticle and lipid droplet traits. Lines above the chromosomes indicate previously identified QTLs. Asterisks indicate previously identified cuticle-associated genes, and triangles indicate potential novel candidate genes.

Several SNP markers uncovered by GWAS for cuticle thickness were located near previously identified cuticle-associated genes in cucumber (Figure 4, Table 3). The most significant SNP, S1_17159027, fell within the gene *SHN1/WIN1* (CsGy1G018900), encoding an ethylene-responsive transcription factor (ERF), which regulates cuticle biosynthesis in many systems, including cucumber [1,3,8,17,18]. S1_17159027 causes a change from leucine to proline within the highly conserved C-terminal CMV-2 motif characteristic of SHN1/WIN1 ERFs [32]. Although not identified as significant by GWAS, all accessions carrying the alternate nucleotide at SNP S1_17159027 also carried the alternate nucleotide at an earlier SNP identified in *CsSHN1/WIN1*, at position S1_17159321 [8]. SNPs at two additional positions within *CsSHN1/WIN1* were also present in the core collection but were not included in the GWAS analysis due to their very low frequency. One of the additional SNPs, S1_17159473, was present in only three accessions, two from India and one from South Asia. Two of the three accessions with the alternate nucleotide at S1_17159473 showed markedly thicker cuticles (5.96 and 7.28) relative to the population mean (4.01), suggesting an additional rare allele of *SHN1* that may affect cuticle properties.

While other significant SNPs did not fall within previously identified genes, we observed that varying SNP filtering parameters or samples included in a dataset can modify GWAS results. GWAS results can also differ among programs using the same datasets and models due to the inherent assumptions used to design each program. For example, a difference of 3 Mb was observed for a peak SNP for resistance to Phytophthora fruit rot [33] when using GAPIT 3.0 vs. rMVP [34], possibly due to differences in algorithms or the presence of multiple tightly linked contributing factors, as was observed when dissecting a QTL for cucumber downy mildew [35]. When we compared GAPIT 3.0 and rMVP for the cuticle and lipid droplet traits, SNPs deemed significant contributors to trait phenotype often fell in similar regions, but with discrepancies in specific locations, typically falling within ~1 Mb. As these observations may suggest support for a genomic region of importance to cuticle and lipid droplet traits, rather than a specific SNP location, we searched for previously identified cuticle-associated genes that were close to GWAS-identified significant SNPs. SNP S1_16881388 for cuticle thickness is located approximately 0.44 Mb away from the *CsWAX2* (*CsGy1G018670*) gene, which influences alkane production in cutin and wax biosynthesis in cucumber, and SNP S5_29662338 is located approximately 0.43 Mb away from the *CsCER6* (*CsGy5G024720*) gene, which influences wax accumulation in cucumber fruit and makes fruit less glossy [14].

### 2.3. GWAS Analysis of Lipid Droplet Traits

**Figure 5:**
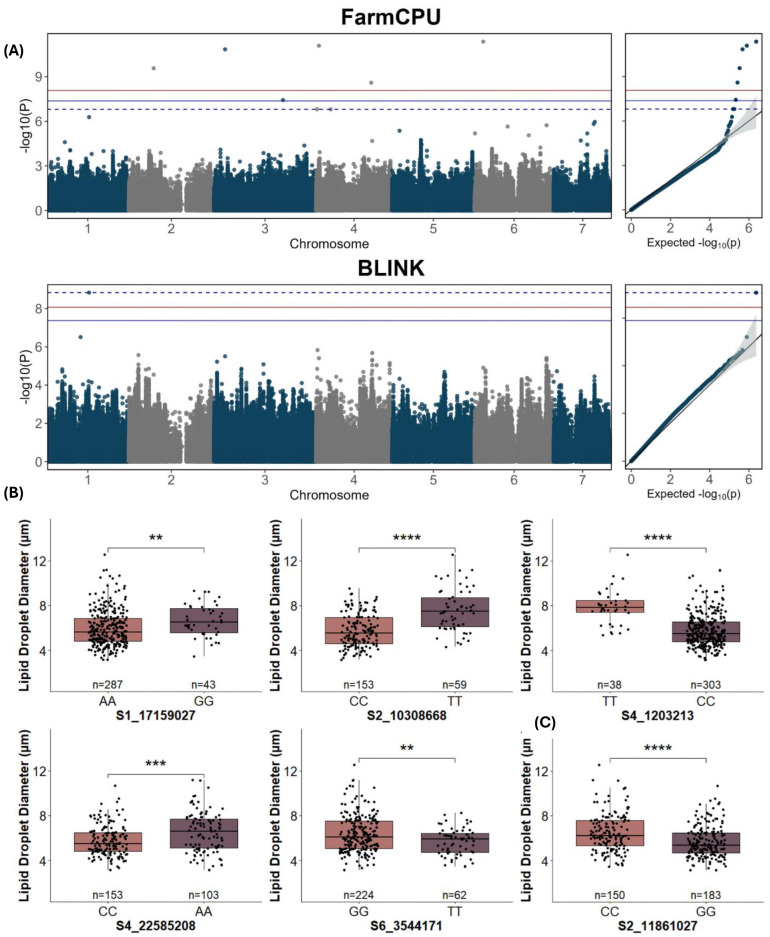
Manhattan, QQ, and allele effect plots for lipid droplet diameter for fruit from the cucumber core collection using BLUE values. (**A**) FarmCPU and BLINK models of GWAS. The blue and red lines represent Bonferroni-corrected *p*-values of 0.05 and 0.01, respectively; the dashed blue line represents FDR ≤ 0.05. (**B**) SNP markers with significant allelic effects. (**C**) Example of the alternate allele effect of SNP within *CsGy2G011870*. BLUE values were calculated from combined data from 2019–2021. **, ***, and **** represent *p* ≤ 0.01, 0.001, and 0.0001, respectively.

**Figure 6:**
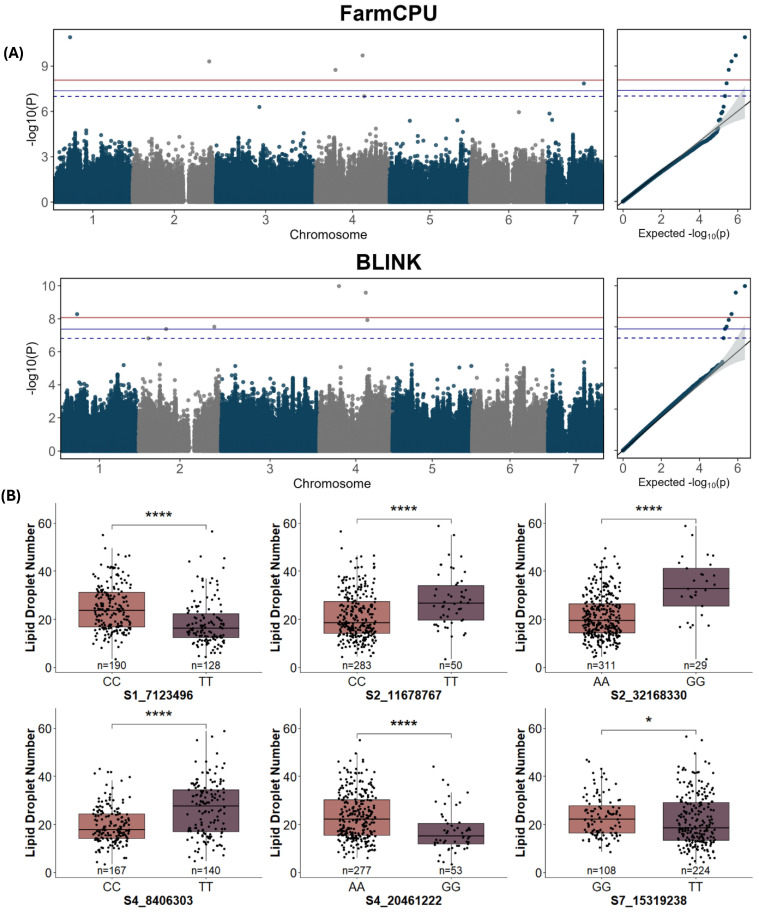
Manhattan, QQ, and allele effect plots for lipid droplet number for fruit from the cucumber core collection using BLUE values. (**A**) FarmCPU and BLINK models of GWAS. The blue and red lines represent Bonferroni-corrected *p*-values of 0.05 and 0.01, respectively; the dashed blue line represents FDR ≤ 0.05. (**B**) SNP markers with significant allelic effects. BLUE values were calculated from combined data from 2019–2021. * and **** represent *p* ≤ 0.05 and 0.0001, respectively.

For lipid droplet traits, 14 QTLs were detected, seven for lipid droplet diameter (Figure 5A, Table 2), and seven for lipid droplet number (Figure 6A, Table 2) using FarmCPU and BLINK models of GWAS at a Bonferroni-corrected threshold of α = 0.05. Five of seven significant SNPs were identified by multiple models for lipid droplet diameter at FDR ≤ 0.05, and all seven significant SNPs were identified by multiple models for lipid droplet number. A significant allelic effect was observed for five of the seven SNPs for lipid droplet diameter (Figure 5B) and for six of the seven SNPs for lipid droplet number (Figure 6B).

Several of the lipid droplet-associated SNPs were located near previously identified QTLs or candidate genes influencing lipid droplets, fruit glossiness, or cuticle permeability. SNP S1_17159027, located on chromosome 1 for lipid droplet diameter, coincided with the SNP for cuticle thickness within the *CsSHN1* gene (Table 3, Figure 4). This SNP accounted for 5.83% of the phenotypic variance observed for lipid droplet diameter, which was one of the larger values for SNPs identified for this trait (Table 2). SNP S2_10308668 is adjacent to a previously identified QTL region for lipid droplet diameter, *qDLD2.1* [8]. SNP S3_28187174 for droplet diameter was located ~0.1 Mb away from the previously identified *CsFSG1/CYP86B1* gene (Figure 4, Table 3), involved in regulating fruit skin gloss [11]. SNP S6_3544171 was located approximately 2Mb away from *CsCER1*, which is associated with alkane biosynthesis and cuticle permeability [12]. 

Consistent with the lack of correlation with cuticle thickness, none of the lipid droplet number QTLs were located in or near previously identified cuticle-associated genes or the SNPs identified for cuticle thickness. However, an SNP for lipid droplet number, S4_8406303, was located within a previously identified QTL region for this trait (*qNLD4.1*) (Figure 4, Table 3) [8]. The QTL region, *qDLD2.1* [8], was flanked by SNPs S2_11678767 for lipid droplet diameter and S2_10308668 for lipid droplet number, further suggesting a factor regulating lipid droplets in this region.

### 2.4. Potential Novel Candidate Genes Associated with Cuticle and Lipid Droplet Traits

To identify new potential candidate genes of interest, we also investigated significant SNPs that were located within genes or within 2.5 kb upstream of an annotated gene; we secondarily asked if the gene was preferentially expressed in fruit peel. For cuticle thickness, two additional ethylene-transcription factors, including a *WIN-1*-like gene, were identified on chromosomes 2 and 5, respectively (SNPs S2_1208493 and S5_29662338) (Table 4A). However, the expression levels for these genes in fruit peel are quite low, as indicated by the fruit development study of [36] and the expression atlas of [37] (accessed from CuGenDB; http://cucurbitgenomics.org/v2/). 

Six genes were identified in close proximity to significant SNPs for lipid droplet number, two of which, *CsGy1G011330* and *CsGy4G009920,* associated with SNPs S1_7123496 and S4_8406303, respectively, showed preferential expression in fruit peel [36,37] (Table 4). S1_7123496 is located within the 3′ UTR region of *CsGy1G011330*, which is predicted to encode a receptor-like serine/threonine-protein kinase. Transcriptome analyses indicate that *CsGy1G011330* shows increasing expression in the peel during the first two weeks of fruit growth ([36,37,38]; transcription data accessed from CuGenDB) are consistent with the increase in lipid droplets and increased expression of other lipid- and cuticle-associated genes observed during that stage of fruit development [7,8].

SNP S4_8406303 is located within a previously identified lipid-droplet associated QTL region on chromosome 4, *qNLD4.1* [8], and is approximately 2 kb upstream of *CsGy4G009920*, a gene that is expressed in fruit peel at a 1000-fold greater level than in fruit flesh (Table 4A, Supplementary Figure S2; [36,37]). This gene also showed a 10–100-fold increase in expression in the peel during early fruit development (Supplementary Figure S2; [36,37,38]). *CsGy4G009920* is predicted to encode an AAI (alpha amylase inhibitor) domain protein containing a plant lipid transfer protein/nonspecific lipid transfer protein type 2 (LTP_2) domain characteristic of AAI proteins [39].

Although three lines of evidence suggested a factor for lipid droplets at ~11 Mb on chromosome 2 (QTL *qDLD2.1* and significant SNPs for lipid droplet diameter and lipid droplet number), there were no previously identified candidate genes in this region. Additionally, we did not identify a candidate gene based on the specific significant SNP locations identified by GWAS. To further examine this region, we took advantage of cucumber transcriptome data and visualization tools [CuGenDB; (http://cucurbitgenomics.org/v2)] and scanned annotated genes located between 10.0 and 12.0 Mb for preferential expression in fruit peel. One gene, *CsGy2G011870*, located at 11.8 Mb, met these criteria, showing the highest expression in fruit peel over a wide range of tissues tested (PRJNA312872 [36]) and ~10× higher expression in fruit peel than in mesocarp (PRJNA448682 [37]) (Table 4B). *CsGy2G011870* encodes a long-chain acyl-CoA synthetase 2, which has been shown to be involved in lipid metabolism in other species [40]. Numerous SNPs located within *CsGy2G011870* showed a highly significant effect for lipid droplet diameter and/or number (e.g., Figure 5C).

## 3. Discussion

The cuticle acts as an important barrier for fruit against desiccation, pests, and pathogens [1,2,3]. It also functions to provide mechanical support throughout fruit development, including the post-harvest stage, with implications for the shelf life and shipping and handling practices of fruits [4,5]. The composition of the cuticle impacts external fruit quality and influences consumer preferences. Many genes involved in the cuticle biosynthetic pathway have been identified, but the factors regulating the natural diversity that exists in populations are largely unknown. In this study, cuticle thickness and lipid droplet traits were characterized in the cucumber core collection. There was extensive variation among all three traits measured as well as geographical differences for the traits. North American accessions tended to have larger lipid droplets and more of them, while accessions from India/South Asia, Central/West Asia and Turkey tended to have smaller lipid droplets and fewer of them.

Normal distribution of the cuticle and lipid droplet traits and identification of multiple QTLs naturally occurring in the cucumber core collection for each trait indicate quantitative inheritance. The low percentage of phenotypic variance explained by many of the significant SNPs highlights the complexity of these quantitative traits. Using multiple models can overcome the limitations associated with each model to help uncover the complex genetic architecture of these traits. Therefore, replication among models and tools can reduce the number of false positives and negatives in GWAS results and increase the validity of significant SNPs [41,42]. It should also be noted that, although the plant materials originated from diverse locations and environments, all accessions were grown in the same location for these experiments. It is possible that some might exhibit somewhat different phenotypes in their native environments and therefore might show different QTLs in those conditions.

Both single-locus and multilocus GWAS statistical models were examined for cuticle and lipid droplet traits. This study focused on the use of multilocus models, such as FarmCPU and BLINK, due to computational efficiency and detection power, along with quantile-quantile (QQ) plots that demonstrated good control of false positive and negative SNP associations [43]. While quite a few SNPs were detected by both models, several SNPs were detected by only one model, demonstrating that SNPs of value can be missed by using only one method. Several studies (e.g., [42,44,45]) have indicated that Bonferroni correction may be overly stringent for GWAS analyses, leading to false rejection of relevant SNPs, in part due to the assumption that SNPs are independent of each other, which may not be the case. When using FDR ≤ 0.05, the majority of SNPs were detected by multiple models, increasing confidence in the identified SNPs. 

### 3.1. GWAS Support for Previously Identified Epidermal Associated QTLs/Genes

The QTLs identified for cuticle and lipid droplet traits were in proximity to several previously identified cuticle-associated QTLs and genes. Chromosome 1 had multiple QTLs identified by GWAS for cuticle thickness and lipid droplet diameter. SNP S1_17159027 was identified as allelically significant for both cuticle thickness and lipid droplet diameter. This SNP is located within the *CsSHN1/WIN1* gene, encoding a transcription factor associated with cuticle biosynthesis regulation [1,3]. A second SNP within *CsSHN1/WIN1*, S1_17159321, was not significantly associated with either trait in the GWAS analysis, but has previously been categorized in cucumber as influential in the natural variation observed in cuticle thickness and lipid droplet diameter for a biparental recombinant inbred line population [8]. All the accessions in the cucumber core collection that had the variant nucleotide at SNP S1_17159027 also had the variant nucleotide at S1_17159321, suggesting a possible functional relationship between the two SNPs. A third, rare, *CsSHN1/WIN1* SNP present in the core collection, S1_17159473, appears to be associated with increased cuticle thickness, indicating the potential value of diversity panels to capture novel alleles. Identification of multiple alleles within *CsSHN1/WIN1* suggests that this gene may be a common target for selection, influencing cuticle-associated traits.

A second significant SNP on chromosome 1, SNP S1_16881388, was located near the *CsWAX2* gene, a gene that impacts alkane production during wax biosynthesis [13]. No significant SNPs were detected within the *CsWAX2* gene itself. Toward the distal end of chromosome 5, there are several simply inherited genes for fruit epidermal features, some of which are tightly linked, including *Heavy/no netting* (*H/h*), *Warty/smooth fruit* (*Tu/tu*), *Dull/glossy fruit skin* (*D/d*), *Mottled/uniform immature fruit color* (*U/u*), and *Tough/tender fruit* (*Te/te*) [46,47], and *CsSEC23* [16]. Interestingly, recent studies have shown that the *Tu*, *D*, and *U* loci are due to a 4895 bp deletion that includes the full sequence of a Zn finger transcription factor gene regulating cuticular wax biosynthesis [19,20,48,49,50]. In the present study, no significant association of any SNPs was identified with these genes. One possible reason is that the causal polymorphism at the *u/Tu/DH’* locus is a large deletion that is absent from the SNP set for GWAS. On the other hand, also on chromosome 5, a significant SNP for cuticle thickness was located 0.43 Mb from *CsCER6*, which regulates fruit cuticular wax accumulation [16]. However, there were no SNPs for *CsCER6* contained in the SNP dataset used to analyze the traits of interest, as the SNPs in this gene were too rare and were filtered out by the SNP filtering parameters used. 

In contrast to cuticle production, lipid droplet traits have far fewer identified genes associated with the regulation of size and number. In Arabidopsis, SEIPIN proteins and OBAP1 have been shown to influence lipid droplet number and size in seeds [26,27]. In cucumber, there are no *SEIPIN* or *OBAP1* homologs near the various QTLs identified by GWAS. Although, as in Arabidopsis, homologs of *OBAP1* (*CsGy7G012610*), *SEIPIN1* (*CsGy1G031230*), and *SEIPIN2* (*CsGy6G035160*) are expressed in cucumber seeds, *OBAP1* and *SEIPIN1* are not expressed in cucumber fruit, and *SEIPIN2* is modestly expressed in fruit peel but at equivalent levels to fruit flesh (transcriptome data accessed from CuGenDB; http://cucurbitgenomics.org/v2). However, a significant QTL for lipid droplet diameter was in close proximity to the fatty acid ω-hydroxylase family gene *CsFSG1/CsCYP86B1* affecting cuticle composition on chromosome 3 [10,11]. On chromosome 2, a QTL previously identified from a biparental study, *qDLD2.1* [8], is flanked by SNPs identified by GWAS for both lipid droplet number and size.

### 3.2. Potential Novel Candidate Genes Identified by GWAS

Several novel candidate genes with significant SNPs, located either within the gene or in the 2.5 kb upstream regulatory region, were identified by GWAS for cuticle and lipid droplet traits. Of particular interest was *CsGy4G009920*, a gene encoding an AAI domain-containing protein identified for lipid droplet number that is located within a previously identified QTL for lipid droplet number [8]. The protein encoded by *CsGy4G009920*, like other AAI proteins, includes a nonspecific lipid transfer protein domain [39]. Multiple functions have been attributed to plant nonspecific lipid transfer proteins, including the secretion of cutin and wax monomers to the cuticles of epidermal cells [51,52]. In a separate study, lipid droplets were isolated for proteomic analysis from the fruit peel of two cucumber cultivars, ‘Gy14’ and ‘Poinsett 76’, harvested at 16 dpa [53]. The AAI protein was observed in samples from both cultivars, further supporting the association of this protein with lipid droplets. 

Examination of numerous plant tissues indicates that *CsGy4G009920* is nearly exclusively expressed in fruit peel (Supplementary Figure S2; [37]). Multiple additional transcriptomic studies (accessed from CuGenDBv2) support the observation of a much higher expression level of *CsGy4G009920* in fruit peel vs. flesh and show the developmental patterns of gene expression that have been observed in other cuticle- and lipid-associated genes [7,8]. Peak expression occurred toward the end of rapid fruit expansion (14–16 dpa). The only tissue other than fruit peel where a high level of expression was observed was tendril. While coiling is a critical function characteristic of tendrils, a second feature shared by tendrils of some species, including members of the Vitaceae, Passifloraceae, Bignoniaceae, and Cucurbitaceae, is adhesion [54]. The mechanisms of attachment vary among families, and to our knowledge have not been determined in cucurbits, but there is precedence for cutin and lipids forming an adhesive fluid in passionflower (*Passifloraceae discophora*) tendrils [55]. A transcriptomic study of cucumber tendril coiling indicated increased expression of transmembrane transport genes during the stretch and coiling stages; expression of *CsGy4G009920* (*CsaV3_4G010460* in CLv3) peaked at the stretch stage [56]. Collectively, the location of the SNP upstream of the start codon, the of the gene with a previously identified lipid droplet QTL [8], the proposed gene colocalization function in lipid transfer, location and timing of expression, and the association of the AAI protein with isolated lipid droplets make *CsGy4G009920* a promising candidate factor influencing lipid droplet accumulation in cucumber fruit peels.

An additional gene of interest is *CsGy2G011870*, which encodes a long-chain acyl-CoA synthetase 2 (LACS2). Multiple functions have been associated with long-chain acyl-CoA synthetases, including the synthesis of membrane lipids and the provision of acyl-CoA pools for cuticle formation [40]. In Arabidopsis, LACS2 is essential for normal cuticle development, and expression of *AtLACS* genes has been found to be positively regulated by cuticle-associated transcription factors, including SHINE1/WIN1 [57,58,59]. The *CsGy2G011870*-encoded LACS gene was found in lipid droplet samples from ‘Poinsett 76’ but not from ‘Gy14’, possibly due to the lower expression levels for this gene relative to the AAI-encoding gene (Table 4). Expression of *CsGy2G011870* in 16 dpp fruit peel was 2-fold lower in ‘Gy14’ than in ‘Poinsett 76’ (PRJNA 345040 [60]). 

## 4. Materials and Methods

### 4.1. Plant Materials and Growth Conditions

Accessions in the cucumber core collection were previously advanced through single seed descent for 2–3 generations to reduce heterozygosity and heterogeneity [30,33]. The list of accessions tested in this study (n = 374) is provided in Supplementary Table S1. Portions of the core collection [29] were grown in triplicate under field conditions at Michigan State University Horticulture and Research Center in 2019–2021. The number of accessions varied each year according to seed availability. A set of 50 accessions was grown in all three years to assess the reproducibility of traits across seasons. The plants were grown on raised black plastic mulch in a randomized complete block design using three replicates with 0.45–0.6 m between plants. Field conditions were as described by Lin et al. [33].

### 4.2. Sample Preparation and Microscopy

**Figure 7:**
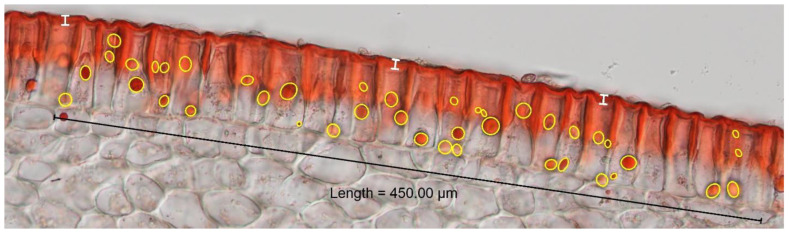
Cross section illustrating measurement of cuticle and lipid droplet traits. Images were taken of cross sections and viewed at 200× magnification, and a uniform line of 450 µm was drawn across each sample. Within this area, the total number of lipid droplets was counted, the area of each lipid droplet was measured (yellow ellipses), and the diameter was calculated using the Nikon NIS-Elements BR software (version 5.30.03). Cuticle thickness was measured in three locations (white lines).

Ovaries were tagged at anthesis, and three fruits were harvested from each accession at 16–20 days post-anthesis (dpa). Fresh tissue samples were prepared from the midsection of the fruit using a sliding block microtome cutting samples to ~0.1 mm thickness. Sections were stained using Sudan IV (Sigma-Aldrich, St. Louis, MO, USA) according to Buda et al. [61] and mounted in glycerin (Columbus Chemical Industries, Columbus, WI, USA). Images were obtained using a Nikon Eclipse Ni-U microscope and Nikon DS-Fi3 camera (Nikon Instruments Inc., Melville, NY, USA) at 200×. For each image, a 450 µm line was drawn, the number of lipid droplets in this area was counted, and the area and diameter of each droplet were measured using Nikon NIS-Elements BR (version 5.30.03) (Figure 7). Measurement of cuticle thickness was made at three locations for each sample image. Best linear unbiased estimates (BLUEs) were obtained for all traits using R package “lme4” [62] and used in subsequent GWAS. The mean square values of each source of variation were used to calculate the genotypic variance *σ*^2^*G* and phenotypic variance *σ*^2^*P*, and broad-sense heritability was calculated using the formula *H*^2^ = *σ*^2^*G/σ*^2^*P*.

### 4.3. GWAS

Cucumber core resequencing data and the previously identified SNP dataset, consisting of 2.5 million SNPs, were downloaded from the Cucurbit Genomics Database (CuGenDB, http://cucurbitgenomics.org/v2/) [30]. GWAS was performed using SNPs that were filtered using BCFtools (version 1.9.64) [63] and VCFtools (version 0.1.15) [64] for the following criteria: biallelic, GQ scores > 20, maximum read depth within two standard deviations of the mean read depth, and minor allele frequency (MAF) > 0.1, resulting in 1,179,473 SNPs for association analysis. Combining genotype and phenotype data from accessions (n = 367), GWAS was performed using multiple models (GLM, MLM, MLM, FarmCPU, and BLINK) implemented with three principal components in the “GAPIT 3.0” software [31] in R (version 4.2.2). Significance thresholds were calculated based on Bonferroni correction for adjusted *p*-values of 0.05 and 0.01 and a false discovery rate (FDR) of 0.05, using the statistical package of Storey et al. (version 2.34.0) [65]. The allele effect was tested by comparing the mean phenotypes of individuals with homozygous alleles (*T*-test; Rprogram:ggpubr). Heterozygotes were included in the figure when they exceeded 10% of the population. In some cases, filtering for GQ score led to elimination of reads for >10% of the accessions at a specific nucleotide position. Seven significant SNPs had greater than 10% missing accessions (four for cuticle thickness and three for lipid droplet diameter); in those cases, the allele effect was also tested when including phenotype data for all accessions. All significant SNPs with the filtered dataset showed the same trends of allele effect when all accessions were included (Supplementary Figure S3).

## 5. Conclusions

The cucumber core collection showed extensive diversity of cuticle and lipid droplet traits in fruit. Using multiple models of GWAS, several SNPs were identified for these traits of interest, spanning the cucumber genome. Many previously identified cuticle-associated genes were located near SNPs identified by GWAS, lending further support to the role of these genomic regions in influencing fruit surface traits. Consistent with multiple prior studies of mutants and biparental populations, the *CsSHN1/WAX1* gene was identified as a candidate gene influencing natural variations in cuticle thickness and lipid droplet diameter. Several novel genes, including a putative lipid transfer protein and a long-chain acyl-CoA synthetase 2, were also implicated for further study.

## Figures and Tables

**Table 1:** Descriptive summary statistics, broad-sense heritability, and correlations of cuticle and lipid droplet traits across the cucumber core population.

Variables	Cuticle Thickness (µm)	Lipid Droplet Diameter (µm)	Lipid Droplet Number
Minimum	1.13	2.06	3.00
Maximum	10.67	12.82	81.00
Mean ^a^	4.01	6.20	22.33
Standard deviation	1.28	2.12	12.62
Fold variation	9.41	6.21	27.00
Broad-sense heritability (H^2^) ^b^	0.45	0.70	0.52
Correlations			
Cuticle thickness		0.46 ***	0.11
Lipid droplet diameter			0.44 **

^a^ Mean value of population (n = 374) for each trait; values for individual accessions are based on measurements from three fruits per accession per year. ^b^ Calculated from a set of 50 accessions that were grown in all three seasons (2019–2021). *H*^2^ = *σ*^2^*_G_/σ*^2^*_P_*. **, *** represent *p* ≤ 0.01, 0.001, respectively.

**Table 2:** Significant SNPs identified in FarmCPU, Blink, MLMM, MLM, and GLM GWAS models (Bonferroni-corrected threshold α = 0.05 and FDR ≤ 0.05) for cuticle thickness and the diameter and number of lipid droplets (DLD, NLD) in the cucumber core collection.

				*p*-Value/Percent Variance Explained (PVE) ^3^				
Trait	SNP Position ^1^	MAF	Alleles ^2^	FarmCPU	BLINK	MLMM	MLM	GLM ^4^
Cuticle Thick.	S1_16881388	0.24	A/C	**3.53 × 10^−08^/2.78 ^5^**	-	-	-	-
	S1_17159027	0.17	A/G	**6.98 × 10^−13^/5.67**	**7.87 × 10^−12^/12.27**	**1.34 × 10^−09^/27.37**	**1.20 × 10^−08^/27.37**	**6.02 × 10^−09^/1/2.96**
	S2_1208493	0.42	G/T	**6.76 × 10^−09^/1.91**	-	-	-	3.13 × 10^−06^
	S4_10360939	0.47	C/T	-	**3.75 × 10^−08^/23.43**	-	-	2.35 × 10^−06^
	S5_29662338	0.32	G/A	**1.42 × 10^−10^/2.56**	-	-	-	5.63 × 10^−06^
	S7_77106	0.22	C/A	**3.85 × 10^−09^/2.05**	-	-	-	-
DLD	S1_17159027	0.17	A/G	-	**1.45 × 10^−09^/5.83**	-	-	-
	S2_10308668	0.37	C/T	**2.69 × 10^−10^/3.40**	-	**4.72 × 10^−09^/33.98**	-	**2.13 × 10^−13^/114/5.79**
	S3_4506374	0.30	T/C	**1.43 × 10^−11^/1.66**	-	-	-	**9.63 × 10^−09^/10/0.04**
	S3_28187174	0.44	C/T	**3.69 × 10^−08^/0.68**	-	-	-	-
	S4_1203213	0.14	T/C	**8.15 × 10^−12^/7.10**	-	-	-	4.71 × 10^−06^
	S4_22585208	0.43	C/A	**2.53 × 10^−09^/1.03**	-	-	-	**1.16 × 10^−08^/6/0.08**
	S6_3544171	0.28	G/T	**4.31 × 10^−12^/1.61**	-	-	-	4.99 × 10^−09^
NLD	S1_7123496	0.42	C/T	**1.22 × 10^−11^/3.37**	**5.22 × 10^−09^/2.55**	-	-	**1.90 × 10^−09^/9/0.59**
	S2_11678767	0.18	C/T	-	**4.18 × 10^−08^/2.59**	-	-	2.98 × 10^−09^
	S2_32168330	0.12	A/G	**4.91 × 10^−10^/8.35**	**3.05 × 10^−08^/4.67**	-	-	**1.09 × 10^−11^/34/1.22**
	S4_8406303	0.46	C/T	**1.79 × 10^−09^/2.13**	**1.05 × 10^−10^/1.97**	-	-	2.54 × 10^−06^
	S4_19786809	0.43	A/G	**1.98 × 10^−10^/2.44**	**2.62 × 10^−10^/2.39**	-	-	2.75 × 10^−06^
	S4_20461222	0.19	A/G	9.97 × 10^−08^	**1.20 × 10^−08^/2.37**	-	-	**2.70 × 10^−08^/1/0.26**
	S7_15319238	0.34	G/T	**1.41 × 10^−08^/3.35**	-	-	-	3.80 × 10^−07^

^1^—Genomic location according to Gy14 v. 2.1 (http://cucurbitgenomics.org/v2/). ^2^—major/minor allele. ^3^—PVE values are reported for SNPs exceeding Bonferroni-corrected threshold (α = 0.05). ^4^—*p*-value for highest SNP in QTL/number of SNPs exceeding FDR ≤ 0.05 in QTL/sum of PVE for SNPs in QTL. ^5^—Bold—SNPs exceeding Bonferroni-corrected threshold (α = 0.05).

**Table 3:** Previously identified cuticle or lipid droplet (LD) associated genes or QTLs in proximity to significant SNPs identified by GWAS.

Trait	SNP Position		Candidate Gene/QTL	Gene ID	Annotation	Distance from SNP	Ref.
	Chr	Bp					
Cuticle thickness	1	16,881,388	*CsWax2*	*CsGy1G018290*	Very-long-chain aldehyde decarbonylase GL1-3	0.44 Mb	[13]
	1	17,159,027	*CsSHN1/WIN1*	*CsGy1G018900*	Ethylene-responsive transcription factor	Within exon	[8]
	5	29,662,338	*CsCER6*	*CsGy5G024720*	3-ketoacyl-CoA-synthase	0.43 Mb	[14]
LD diameter	1	17,159,027	*CsSHN1/WIN1*	*CsGy1G018900*	Ethylene-responsive transcription factor	Within exon	[8]
	2	11,678,767	*qDLD2.1*				[8]
	3	28,187,174	*CsFSG1/CYP86B1*	*CsGy3G027185*	Cytochrome P450, CYP86B1	0.01 Mb	[11]
	6	3,544,171	*CsCER1*	*CsGy6G006240*	Very-long-chain aldehyde decarbonylase GL1-1	1.92 Mb	[12]
LD number	2	10,308,668	*qDLD2.1*				[8]
	4	8,406,303	*qNLD4.1*				[8]

**Table 4:** Novel cuticle and lipid droplet candidate genes. (**A**) Genes in closest proximity to SNPs identified by GWAS (SNP located within the gene or within 2.5 kb upstream of the gene) and (**B**) fruit peel preferentially expressed gene within lipid droplet diameter QTL *qDLD2.1*.

	SNP Position				Expression in Fruit (FPKM) ^1^		
(A) Trait	Chr	Bp	SNP within Gene/<2.5 kb Upstream	Annotation	Flesh (9 dpa)	Peel (9 dpa)	SNP Position Relative to Gene
Cuticle	2	1,208,493	*CsGy2G001760*	Ethylene-responsive transcription factor WIN1-like	0	6	2515 bp upstream
thickness	5	29,662,338	*CsGy5G025410*	Ethylene-responsive transcription factor WRI1	2	5	within intron
LD no.	1	7,123,496	*CsGy1G011330*	Receptor-like serine/threonine-protein kinase	0	19	within 3′ UTR
	2	32,168,330	*CsGy2G024390*	Delta-aminolevulinic acid dehydratase	88	112	within intron
	4	8,406,303	*CsGy4G009920*	AAI domain-containing protein, lipid transfer	4	4894	2111 bp upstream
	4	19,786,809	*CsGy4G015060*	Zinc finger CCCH domain-containing protein 25	12	14	1684 bp upstream
	4	20,461,222	*CsGy4G015760*	Nonspecific serine/threonine protein kinase	95	25	630 bp upstream
	7	15,319,238	*CsGy7G012000*	Pathogen-related protein-like	17	28	within intron
**(B) Trait**	**Chr**	***QTL***	**Candidate Gene**		**Flesh (9 dpa)**	**Peel (9 dpa)**	
LD diameter; LD no.	2	*qDLD2.1*	*CsGy2G011870*	long chain acyl-CoA synthetase 2	8	77	4 SNPs in exons, 1 in 5′UTR

^1^ Expression data from variety Yan Bai, PRNJA448682 [36], accessed from CuGenDB (http://cucurbitgenomics.org/v2).

## Data Availability

All sequence data used for GWAS are available on the CuGenDB (http://cucurbitgenomics.org/v2/). All other data are provided in the manuscript and Supplementary Materials.

## Supplementary Materials

The supporting information can be downloaded at: https://www.mdpi.com/article/10.3390/ijms25179306/s1.
