# Supplementary material for: Genome-Wide Association Study of Cuticle and Lipid Droplet Properties of Cucumber (Cucumis sativus L.) Fruit
Source: Int J Mol Sci. 2024 Aug 28;25(17):9306. doi: 10.3390/ijms25179306 (PMC11395541; doi:10.3390/ijms25179306)
Supplement: Supplementary file 1 [file ijms-25-09306-s001.zip › Supplemental Figures.pdf]

A) Cuticle Thickness

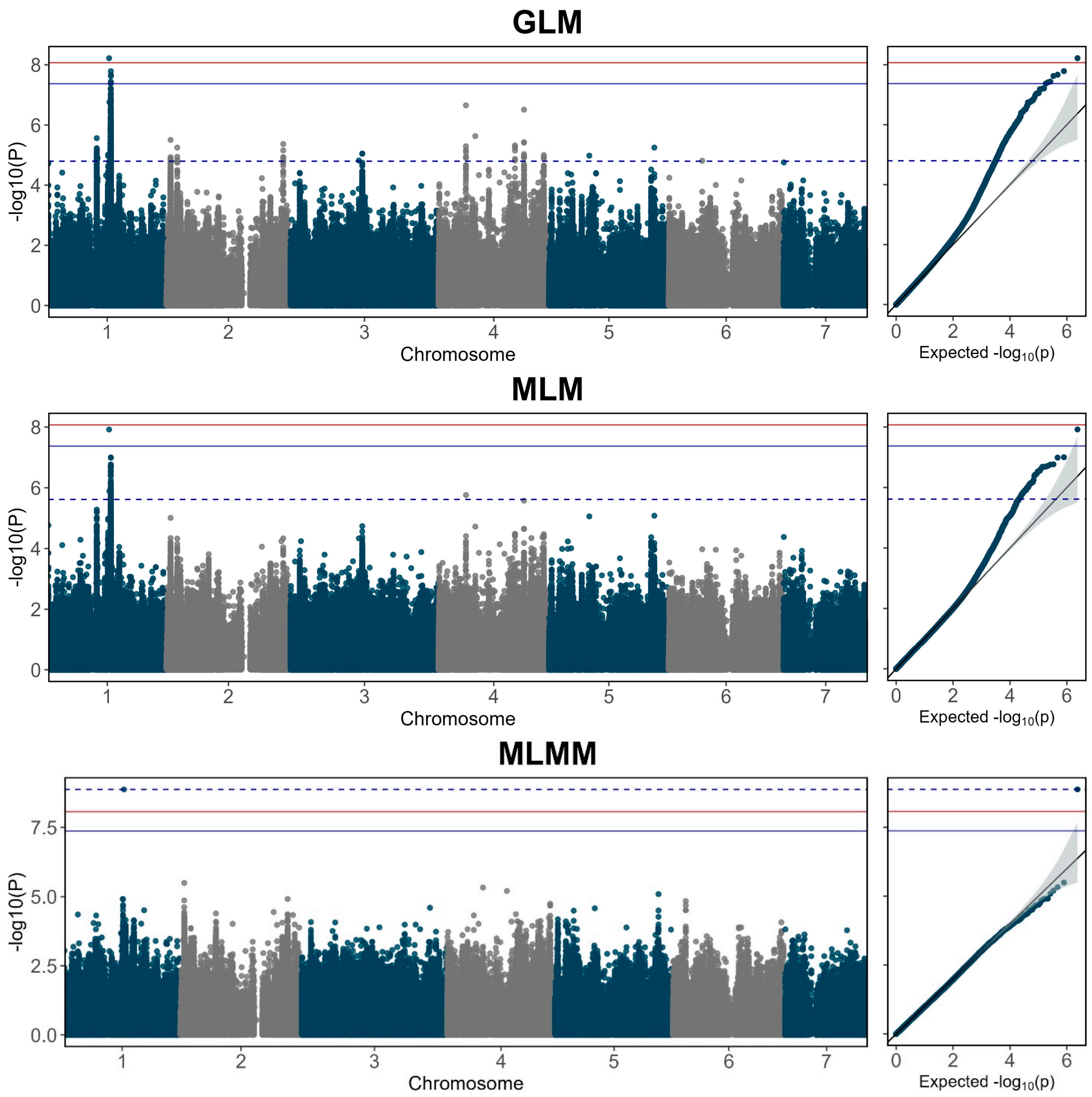

B) Lipid Droplet Diameter

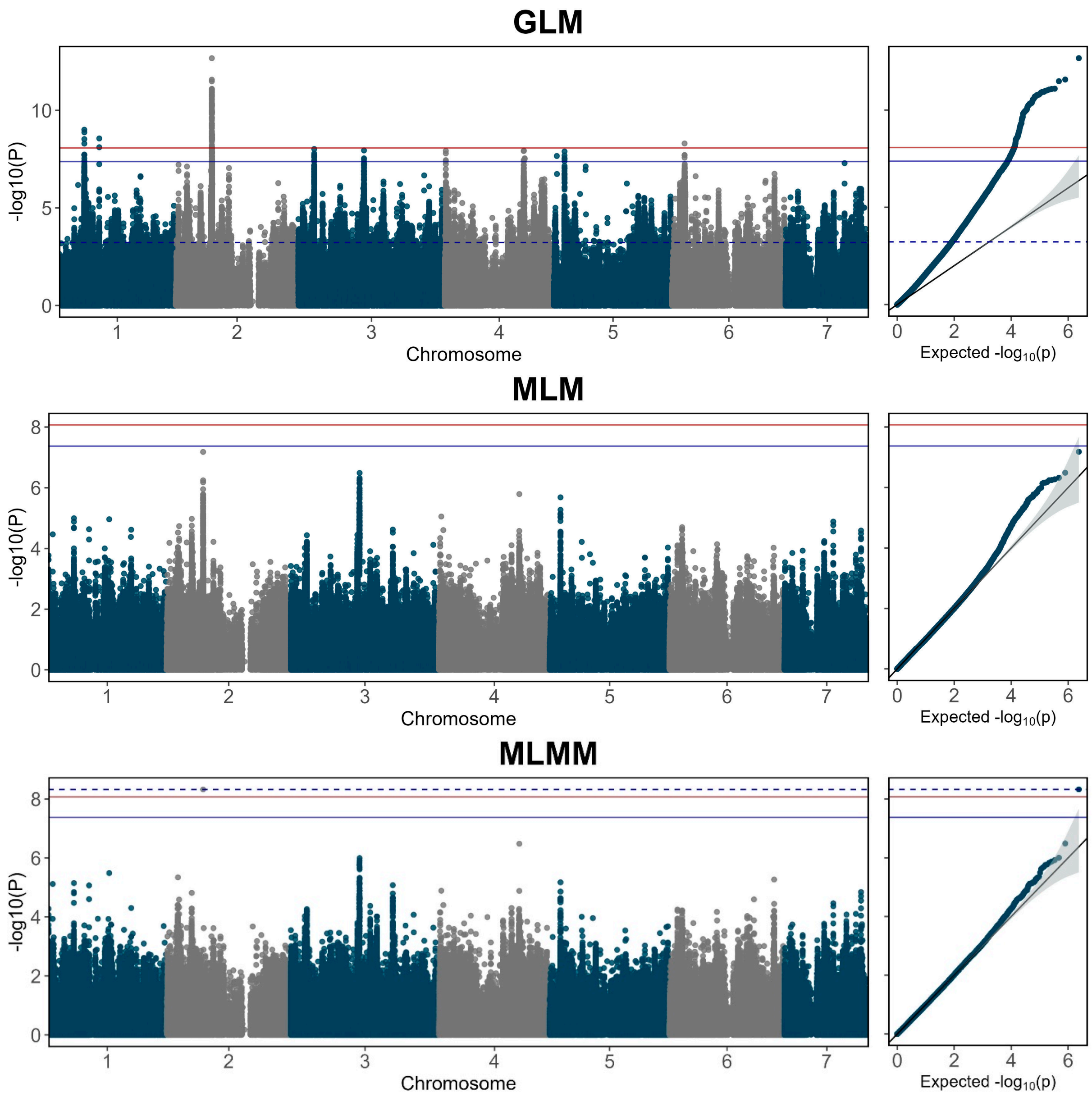

C) Lipid Droplet Number

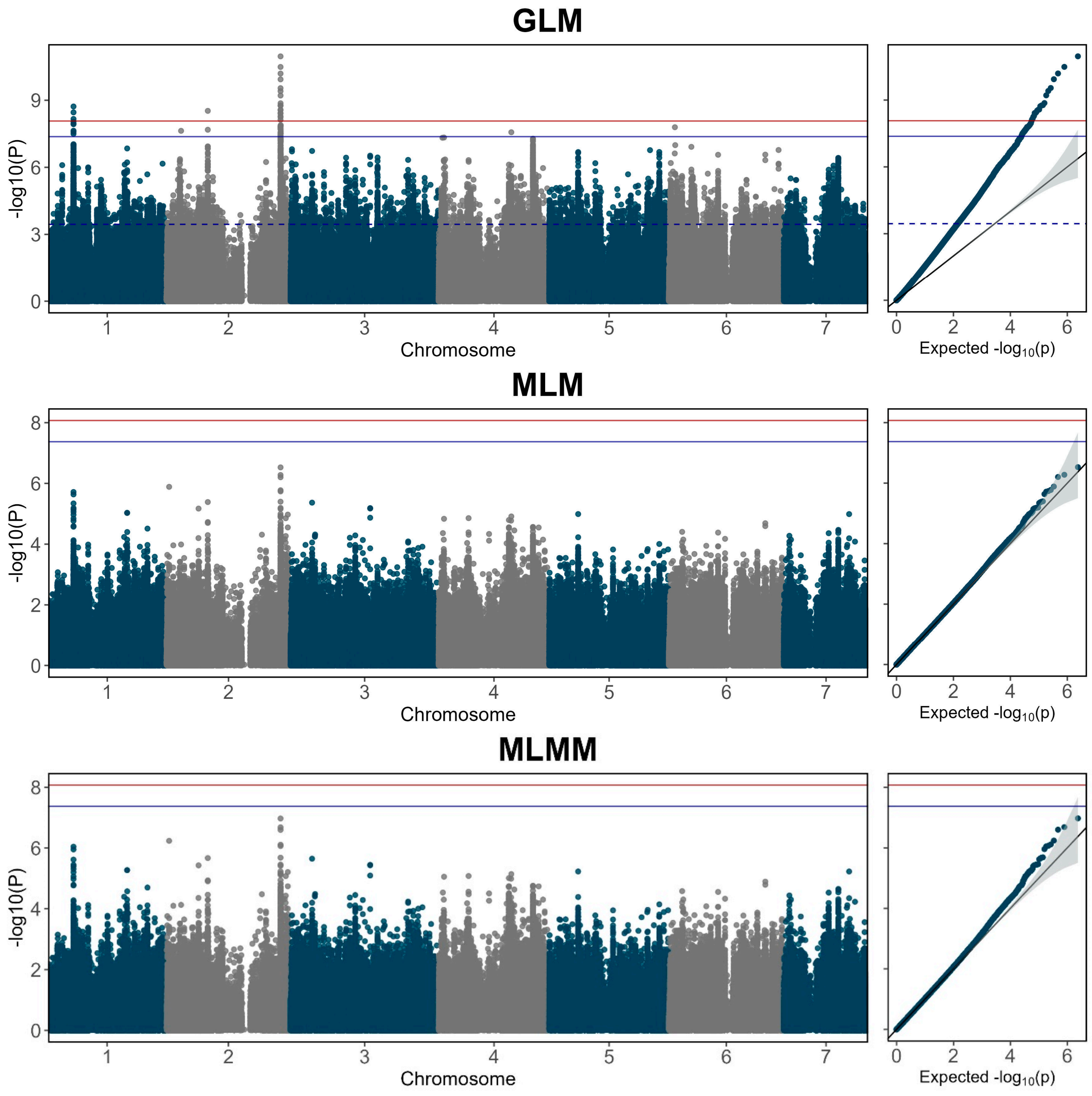

Supplemental Figure S1. Manhattan and QQ plots for cuticle and lipid droplet traits for fruit from the cucumber core collection using BLUE values. GLM, MLM, and MLMM GWAS models for **A)** Cuticle Thickness, **B)** Lipid Droplet Diameter, and **C)** Lipid Droplet Number. BLUE values were calculated from combined data from 2019-2021. The blue and red lines represent Bonferroni corrected p-values of 0.05 and 0.01, respectively; the dashed blue line represents FDR≤0.05.

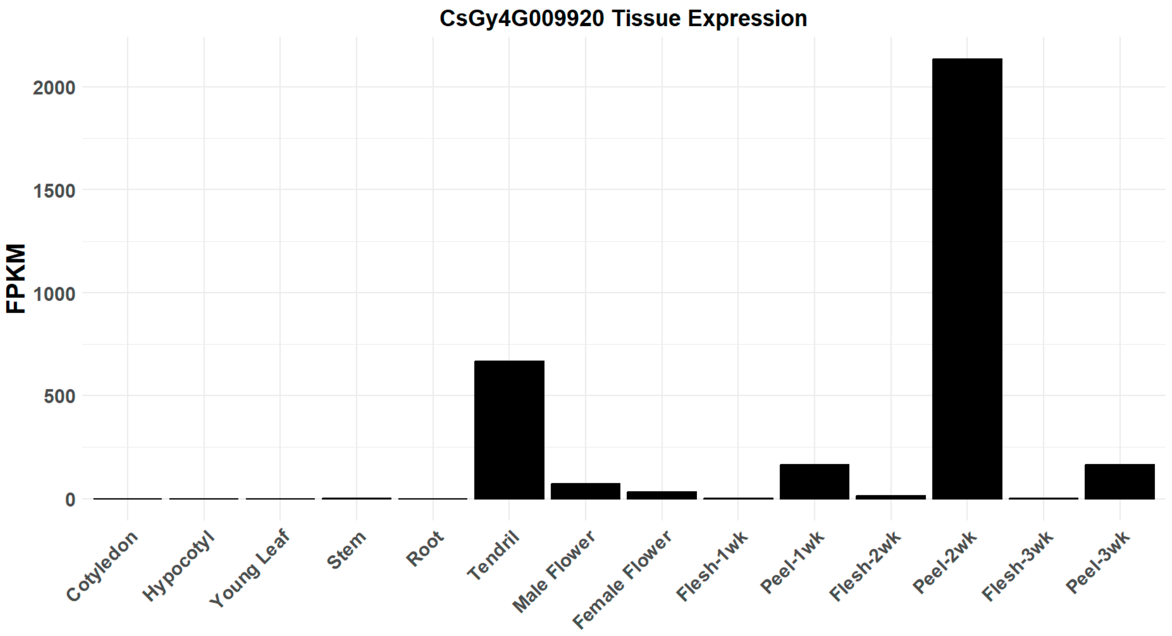

Supplemental Figure S2. Cucumber tissue expression data for *CsGy4G009920.* Transcriptome data were accessed from **PRJNA312872** (Wei et al., 2016) CuGenDB (http://cucurbitgenomics.org/v2).

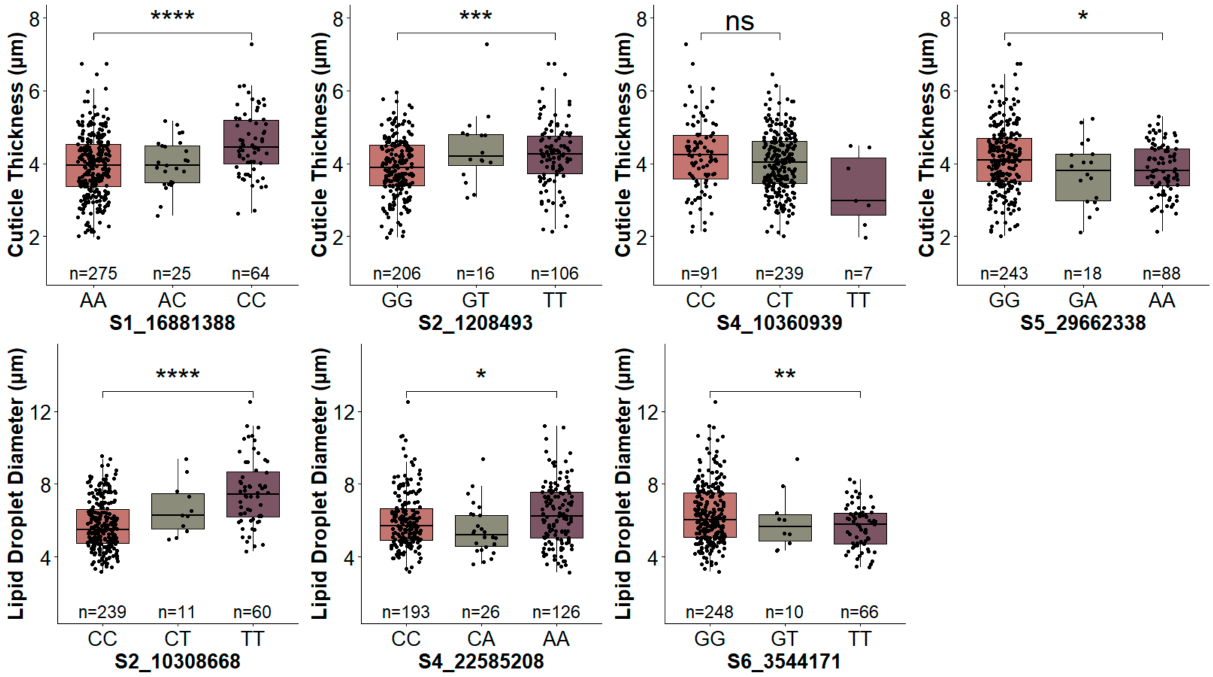

Supplemental Figure S3. Allele effect test of GWAS-identified significant SNPs that had greater than 10% missing accessions due to GQ value of nucleotide call when including all phenotype data for all accessions.
