# Supplementary material for: Genome-Wide Association Study of Cuticle and Lipid Droplet Properties of Cucumber (Cucumis sativus L.) Fruit
Source: Int J Mol Sci. 2024 Aug 28;25(17):9306. doi: 10.3390/ijms25179306 (PMC11395541; doi:10.3390/ijms25179306)
Supplement: Supplementary file 1 [file ijms-25-09306-s001.zip › Supplemental Table S2.pdf]

Supplemental Table S2. Correlations among years for cuticle and lipid droplet traits using 50 accessions grown over all three seasons (2019-2021).

	CT 2020	CT 2021	LDD 2020	LDD 2021	LDN 2020	LDN 2021
**Cuticle Thickness 2019**	0.43**	0.31*				
**Cuticle Thickness 2020**	-	0.46***				
**Lipid Droplet Diameter 2019**			0.81***	0.68***		
**Lipid Droplet Diameter 2020**			-	0.78***		
**Lipid Droplet Number 2019**					0.78***	0.75***
**Lipid Droplet Number 2020**					-	0.82***

Correlations based on mean values on measurements from three fruits per accession per year.
Abbreviations: Cuticle Thickness (CT), Lipid Droplet Diameter (LDD), and Lipid Droplet Number (LDN)
